# Altered Embryonic SIRT6 and SIRT7 Expression Is Associated with Impaired Developmental Competence and Oocyte-Dependent Embryo Quality in Embryos Derived from Subfertile Males

**DOI:** 10.3390/ijms27167115

**Published:** 2026-08-08

**Authors:** Alicja Kowalczyk, Mercedes Camiña García, José Ángel Hernández Malagón, Jose Pedro Araujo, Ivona Žura Žaja, Marko Pećin, Dražen Đuričić, Elżbieta Gałęska, Zbigniew Dobrzański, Marko Samardžija

**Affiliations:** 1 Department of Environment Hygiene and Animal Welfare, Wrocław University of Environmental and Life Sciences, Chełmońskiego 38C, 51-630 Wrocław, Poland; 2 Research and Development Center, Voivodeship Specialist Hospital in Wrocław, 51-124 Wrocław, Poland; 3 Department of Physiology, Faculty of Veterinary, University of Santiago de Compostela, 27002 Lugo, Spain; 4 COPAR (GI-2120) Research Group, Department of Animal Pathology, Faculty of Veterinary, University of Santiago de Compostela, 27002 Lugo, Spain; 5 Mountain Research Centre, Escola Superior Agrária, Instituto Politécnico de Viana do Castelo, 4900-347 Viana do Castelo, Portugal; 6 Department of Physiology and Radiobiology, Faculty of Veterinary Medicine, University of Zagreb, Heinzelova 55, 10000 Zagreb, Croatia; izzaja@vef.unizg.hr (I.Ž.Ž.); ddjuricic@vef.unizg.hr (D.Đ.); 7 Clinic for Surgery, Orthopedics and Ophthalmology, Faculty of Veterinary Medicine, University of Zagreb, 10000 Zagreb, Croatia; mpecin@vef.unizg.hr; 8 Clinic of Obstetrics and Reproduction, Faculty of Veterinary Medicine, University of Zagreb, 10000 Zagreb, Croatia

**Keywords:** animal model, sirtuins, embryos, embryonic developmental competence, paternal effects, oocyte source

## Abstract

Paternal contributions to preimplantation embryo development are increasingly recognized as an important component of reproductive success, but the associations between male reproductive phenotype, early embryonic competence, and embryo molecular characteristics remain insufficiently defined. Using bovine in vitro embryo production as a translational large-mammalian model, this study evaluated whether paternal reproductive phenotype and oocyte origin were associated with embryo developmental performance and embryonic sirtuin expression. Oocytes obtained from mature cows or heifers were fertilized with semen from bulls differing in reproductive status, including bulls characterized by libido disturbances and irregular semen collection. Fertilization, cleavage, blastocyst formation, and hatching were assessed, while embryonic SIRT2, SIRT3, SIRT6, and SIRT7 expression was quantified by immunofluorescence. Early developmental endpoints were relatively preserved, whereas differences were more evident at later stages of preimplantation development. Cow-derived oocytes were associated with higher hatching rates than heifer-derived oocytes. Embryos generated using semen from bulls with an impaired reproductive phenotype showed lower developmental competence and lower SIRT6 and SIRT7 expression. SIRT6 expression showed the strongest positive correlation with hatching, and positive correlations were also observed among the analyzed sirtuins. These findings indicate that paternal reproductive phenotype and oocyte origin are associated with bovine embryo developmental competence and embryonic sirtuin profiles. Embryonic SIRT6 and SIRT7 expression may represent candidate molecular indicators of embryo competence; however, causal relationships cannot be inferred from the present study design.

## 1. Introduction

Although most studies have focused primarily on the evaluation of semen quality, the associations between paternal reproductive phenotype, embryonic molecular characteristics, and preimplantation developmental competence are still not sufficiently understood [1]. Consequently, paternal contributions and sperm-borne epigenetic information are becoming increasingly important areas of research in reproductive biology and embryology [2,3].

Sirtuins are NAD^+^-dependent deacylases involved in the regulation of mitochondrial metabolism, cellular redox balance, cytoskeletal organization, chromatin remodeling, DNA repair, and genome stability. Increasing evidence indicates that individual members of the sirtuin family perform different but complementary functions during gametogenesis, fertilization, and preimplantation embryo development [4,5].

In the present study, SIRT2, SIRT3, SIRT6 and SIRT7 were selected to represent cytoskeletal and cell division-associated, mitochondrial, and nuclear/chromatin-related components of sirtuin-dependent regulation. SIRT2 is particularly relevant to the bovine reproductive model because it is expressed during bovine oocyte maturation and preimplantation embryo development. Experimental inhibition of SIRT2 disrupts spindle organization and chromosome alignment, increases α-tubulin and H4K16, alters organelle distribution, affects mitochondrial function, and disrupts redox homeostasis in bovine oocytes [6]. SIRT3 is predominantly associated with mitochondrial energy metabolism and antioxidant defense, while SIRT6 and SIRT7 are nuclear regulators involved in chromatin organization, genome maintenance, DNA damage responses, and cellular adaptation to stress [4,5]. Therefore, the selected panel was designed to examine functionally complementary sirtuin-associated processes associated with sirtuin that may contribute to embryo competence and its relationship with paternal reproductive status and oocyte origin.

Although SIRT1, SIRT4 and SIRT5 have also been implicated in reproductive physiology, mitochondrial homeostasis, and gamete or embryo competence [4,5,7], they were not included in the predefined analytical panel. The present study was not designed as a comprehensive analysis or ranking of all mammalian sirtuins. SIRT2, SIRT3, SIRT6 and SIRT7 were selected before the analyses to represent complementary cytoskeletal and cell division-associated, mitochondrial and nuclear/chromatin-related processes. Therefore, the exclusion of other members of the sirtuin family should not be interpreted as evidence of their lesser biological relevance.

Moreover, sperm-transmitted paternal epigenetic factors can influence embryonic gene expression and development competence during preimplantation development [8]. However, despite increasing evidence linking sirtuins with reproductive physiology, the relationship between paternal reproductive status, embryonic sirtuin expression, and bovine embryo developmental competence remains largely unexplored. In particular, little is known about whether embryonic SIRT6 and SIRT7 expression is associated with paternal reproductive phenotype and developmental competence during bovine preimplantation development.

The present study aimed to assess whether paternal reproductive status and maternal oocyte origin were associated with the developmental competence of bovine embryos and the embryonic expression of selected sirtuins, namely SIRT2, SIRT3, SIRT6, and SIRT7. Furthermore, we investigated associations between developmental outcomes and embryonic sirtuin expression profiles in embryos derived from bulls with differing reproductive performance and libido status. The study was designed as an associative investigation and was not intended to determine causal or mediating mechanisms.

## 2. Results

### 2.1. Effect of Paternal Reproductive Status and Oocyte Source on Embryo Developmental Competence

**Figure 1:**
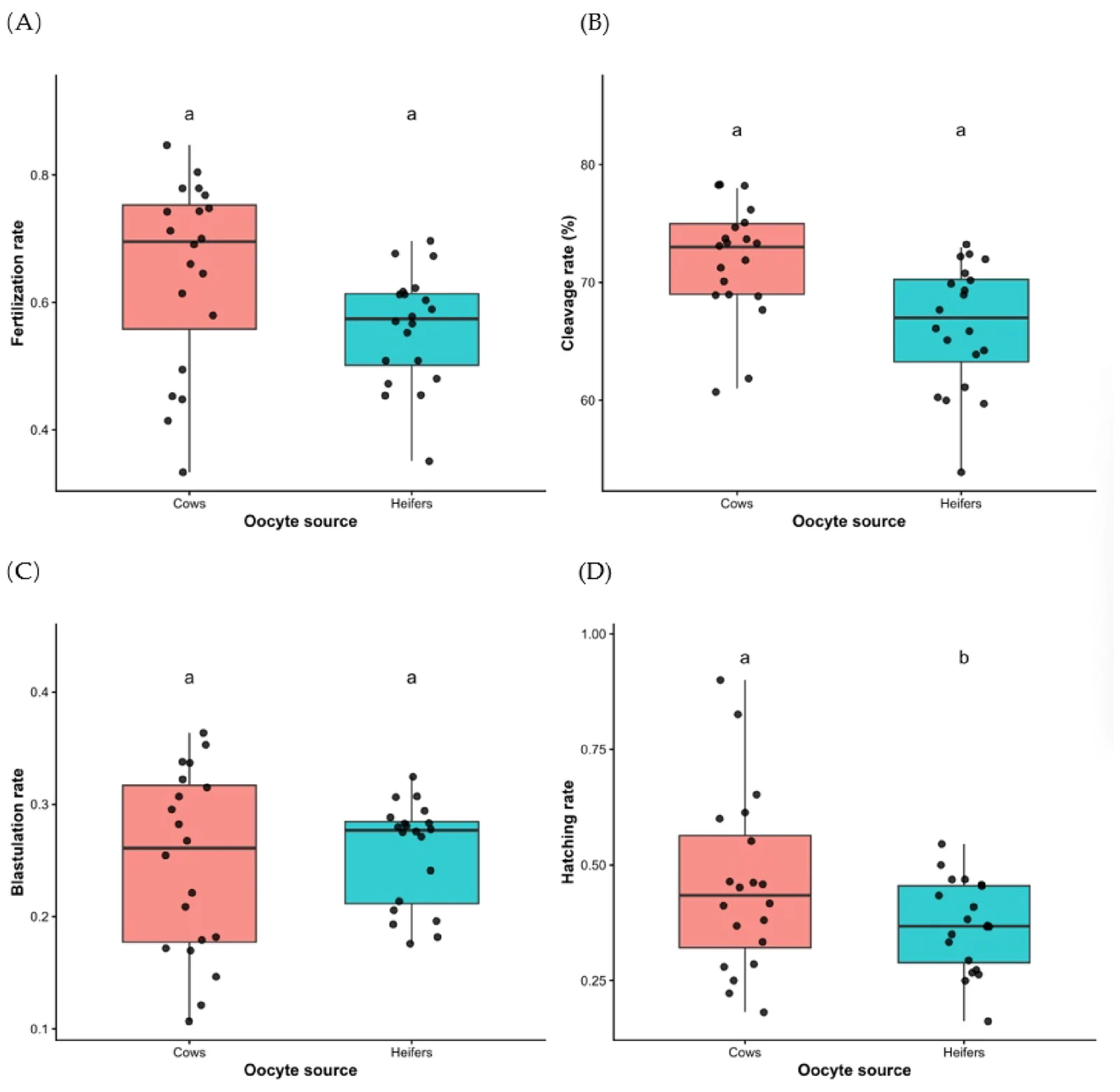
Effects of paternal reproductive status and oocyte source on bovine embryo developmental competence. Fertilization rate assessed on Day 1 (**A**), cleavage rate assessed on Day 3 (**B**), blastocyst formation assessed on Day 8 (**C**), and hatching rate assessed on Day 8 (**D**). Developmental outcomes are expressed as percentages relative to the number of inseminated oocytes. Data are presented as mean ± SD. Statistical analysis was performed using generalized linear mixed models with paternal reproductive group, oocyte source, and their interaction as fixed effects and replicate as a random effect. Pairwise comparisons were performed using Tukey-adjusted estimated marginal means. Different letters indicate significant differences at *p* < 0.05.

Fertilization rates did not differ significantly according to oocyte source, paternal reproductive group, or their interaction (*p* > 0.05). The median fertilization rate was approximately 70% for cow-derived oocytes and 57% for heifer-derived oocytes (Figure 1A). Similarly, cleavage rates assessed on Day 3 did not differ significantly between embryos derived from cow and heifer oocytes (*p* > 0.05). The median cleavage rates were approximately 73% and 67% for cow- and heifer-derived embryos, respectively (Figure 1B).

Blastocyst formation on Day 8 was also comparable between the two oocyte-source groups (*p* > 0.05). The median blastulation rates were approximately 26% for cow-derived embryos and 28% for heifer-derived embryos, although greater variability was observed between embryos originating from cow oocytes (Figure 1C). In contrast, hatching rate differed significantly between the oocyte-source groups (*p* < 0.05). Embryos derived from cow oocytes exhibited a higher hatching rate than those derived from heifer oocytes, with median values of approximately 43% and 36%, respectively (Figure 1D).

Overall developmental competence differed according to paternal reproductive group. Embryos generated using semen from Group III bulls showed lower developmental outcomes than embryos derived from control bulls, while no significant differences were detected between the Control, Group I, and Group II bulls. A significant interaction between Group III and heifer-derived oocytes was also identified, indicating that the association between the paternal reproductive group and the developmental outcome differed according to the source of the oocyte (Table 1).

### 2.2. Embryonic SIRT2, SIRT3, SIRT6, and SIRT7 Expression

Linear mixed-effects model analysis showed that embryonic SIRT2 expression differed significantly according to paternal reproductive group (F(3, 60) = 46.385, *p* < 0.001) and developmental day (F(1, 60) = 64.958, *p* < 0.001), whereas no significant overall association with oocyte source was detected (F(1, 60) = 0.491, *p* = 0.486). A significant paternal reproductive group × oocyte source interaction was detected (F(3, 60) = 4.891, *p* = 0.004), indicating that the magnitude of differences among paternal groups varied according to oocyte origin. The other two-way interactions and the three-way interaction were not significant (all *p* ≥ 0.740). Among embryos derived from cow oocytes, Group II showed higher SIRT2 expression than the Control, Group I, and Group III (estimated marginal means: 0.800, 0.460, 0.450, and 0.360, respectively; all *p* < 0.001). Among embryos derived from heifer oocytes, Group II showed higher expression than the Control (0.710 vs. 0.520; *p* = 0.008) and Group III (0.710 vs. 0.200; *p* < 0.001). Group III also showed lower expression than the Control and Group I (0.200 vs. 0.520 and 0.560, respectively; both *p* < 0.001). SIRT2 expression was higher on Day 3 than on Day 8 (0.623 vs. 0.393; *p* < 0.001).

Embryonic SIRT3 expression differed significantly according to paternal reproductive group (F(3, 60) = 81.706, *p* < 0.001) and developmental day (F(1, 60) = 89.269, *p* < 0.001), whereas no significant overall association with oocyte source was detected (F(1, 60) = 0.508, *p* = 0.479). None of the two-way or three-way interactions reached statistical significance (all *p* ≥ 0.636). Tukey-adjusted comparisons showed that all paternal groups differed significantly from one another (all *p* < 0.001). Group II showed the highest estimated marginal SIRT3 expression, followed by Group I, the Control, and Group III (0.870, 0.645, 0.485, and 0.270, respectively). SIRT3 expression was higher on Day 3 than on Day 8 (0.700 vs. 0.435; *p* < 0.001).

**Figure 2:**
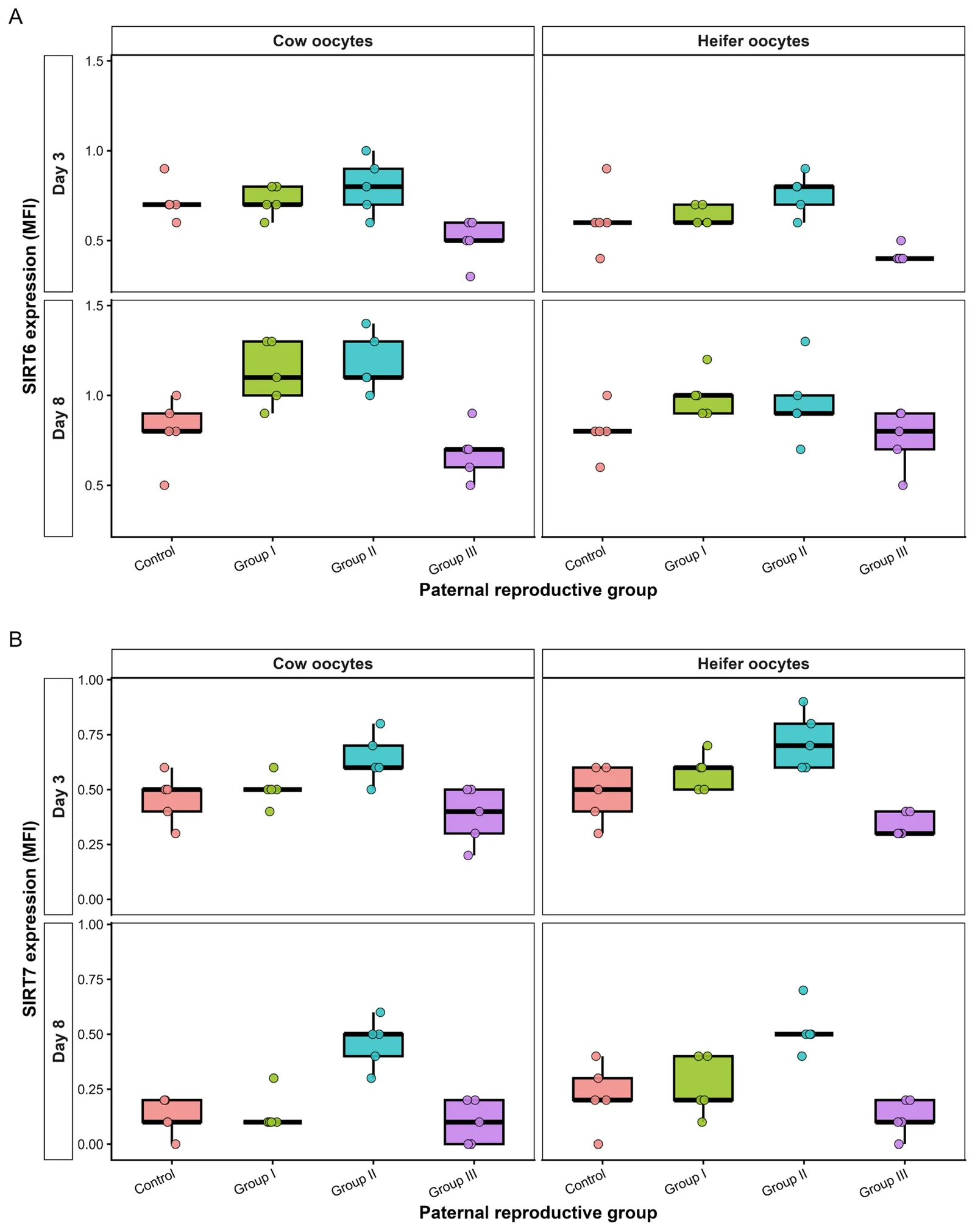
Embryonic SIRT6 (**A**) and SIRT7 (**B**) expression according to paternal reproductive group, oocyte source, and developmental day. Columns represent embryos derived from cow and heifer oocytes, whereas rows represent Day 3 and Day 8 of embryo development. Individual points represent experimental observations. Boxes indicate the median and interquartile range, and whiskers extend to values within 1.5 times the interquartile range. Statistical effects of paternal reproductive group, oocyte source, developmental day, and their interactions were evaluated using linear mixed-effects models, with experimental replicate included as a random intercept. Detailed model results and Tukey-adjusted comparisons are reported in the main text.

SIRT6 expression differed significantly according to paternal reproductive group (F(3, 64) = 21.349, *p* < 0.001), oocyte source (F(1, 64) = 4.667, *p* = 0.035), and developmental day (F(1, 64) = 66.881, *p* < 0.001). None of the analyzed interactions was statistically significant, including paternal reproductive group × oocyte source (*p* = 0.511), paternal reproductive group × developmental day (*p* = 0.064), oocyte source × developmental day (*p* = 0.878), and the three-way interaction (*p* = 0.259). Group II and Group I showed higher SIRT6 expression than both the Control and Group III. The estimated marginal means were 0.925, 0.870, 0.735, and 0.590 for Group II, Group I, the Control, and Group III, respectively. Compared with the Control, expression was higher in Group II (*p* < 0.001) and Group I (*p* = 0.023). Both Group II and Group I also showed higher expression than Group III (both *p* < 0.001). The Control showed higher expression than Group III (*p* = 0.013). SIRT6 expression was higher in embryos derived from cow oocytes than in those derived from heifer oocytes (0.815 vs. 0.745; *p* = 0.035) and was higher on Day 8 than on Day 3 (0.913 vs. 0.648; *p* < 0.001; Figure 2A).

Similarly, SIRT7 expression differed significantly according to paternal reproductive group (F(3, 60) = 47.533, *p* < 0.001), oocyte source (F(1, 60) = 6.465, *p* = 0.014), and developmental day (F(1, 60) = 155.797, *p* < 0.001). The paternal reproductive group × oocyte source interaction (*p* = 0.333), paternal reproductive group × developmental day interaction (*p* = 0.092), oocyte source × developmental day interaction (*p* = 0.359), and the three-way interaction (*p* = 0.861) were not significant. Group II showed the highest estimated marginal SIRT7 expression and differed from the Control, Group I, and Group III (0.585 vs. 0.320, 0.370, and 0.235, respectively; all *p* < 0.001). Group III showed lower expression than the Control (*p* = 0.036) and Group I (*p* < 0.001), whereas the Control and Group I did not differ significantly. SIRT7 expression was higher in embryos derived from heifer oocytes than in cow-derived embryos (0.405 vs. 0.350; *p* = 0.014) and was higher on Day 3 than on Day 8 (0.513 vs. 0.243; *p* < 0.001; Figure 2B).

### 2.3. Associations Between Embryonic Sirtuin Expression and Developmental Outcomes

**Figure 3:**
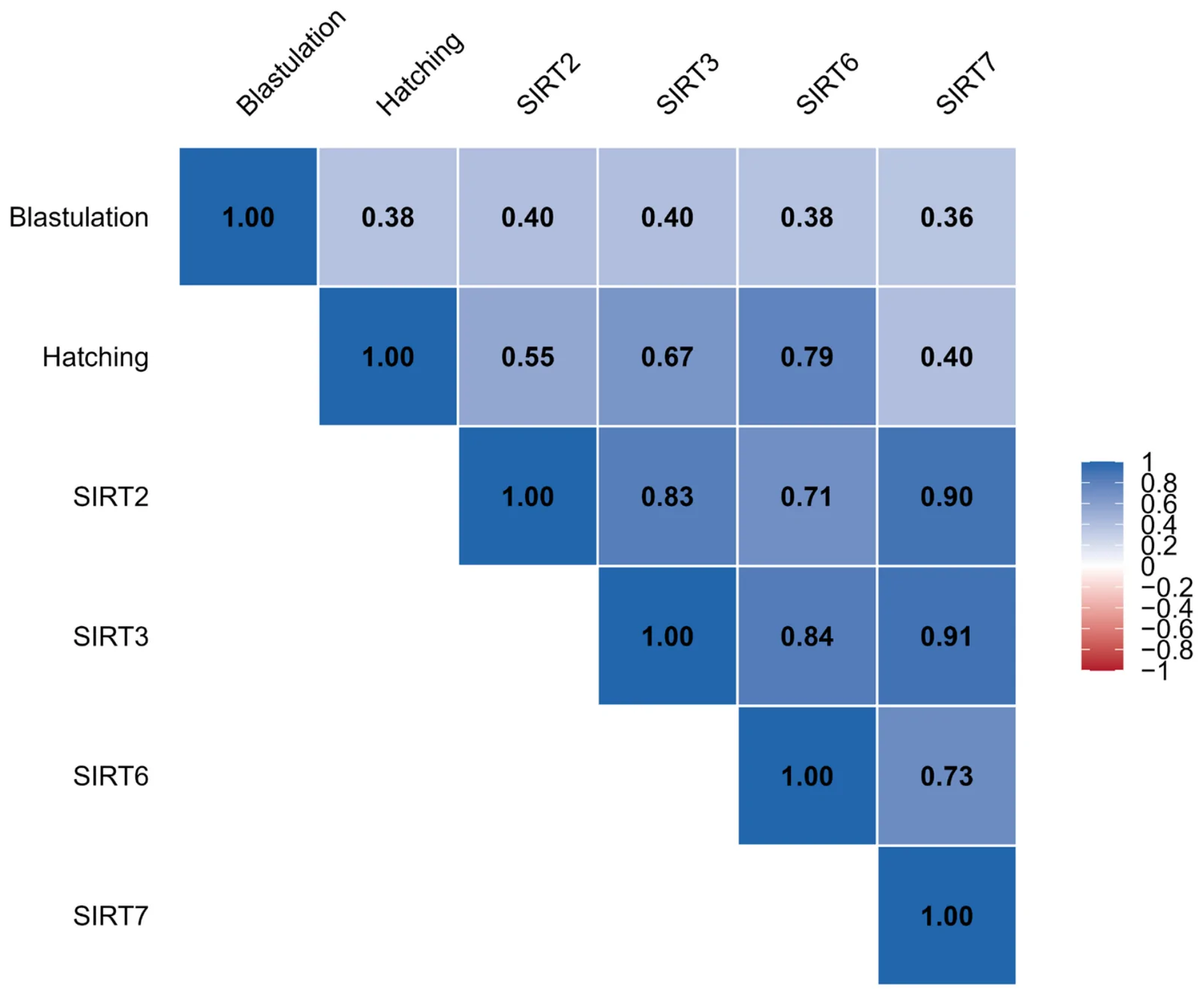
Spearman’s correlation matrix between bovine embryo developmental outcomes and embryonic sirtuin expression. The matrix presents Spearman’s rank correlation coefficients (*ρ*) for fertilization, cleavage, blastulation, and hatching rates and the mean fluorescence intensity of SIRT2, SIRT3, SIRT6, and SIRT7. Positive and negative correlations are indicated by the color scale, with color intensity corresponding to correlation strength.

Spearman’s correlation analysis showed that hatching rate was most strongly associated with SIRT6 expression (*ρ* = 0.79), followed by SIRT3 (*ρ* = 0.67), SIRT2 (*ρ* = 0.55), and SIRT7 expression (*ρ* = 0.40; Figure 3). Associations between blastulation rate and sirtuin expression were weaker and similar in magnitude, ranging from *ρ* = 0.36 for SIRT7 to *ρ* = 0.40 for SIRT2 and SIRT3. Blastulation and hatching rates were positively correlated at *ρ* = 0.38.

Strong positive correlations were also observed among the analyzed sirtuins. The highest coefficients were found between SIRT3 and SIRT7 (*ρ* = 0.91), SIRT2 and SIRT7 (*ρ* = 0.90), and SIRT3 and SIRT6 (*ρ* = 0.84). SIRT6 and SIRT7 expression were correlated at *ρ* = 0.73.

### 2.4. Principal Component Analysis of Embryonic Sirtuin Expression

**Figure 4:**
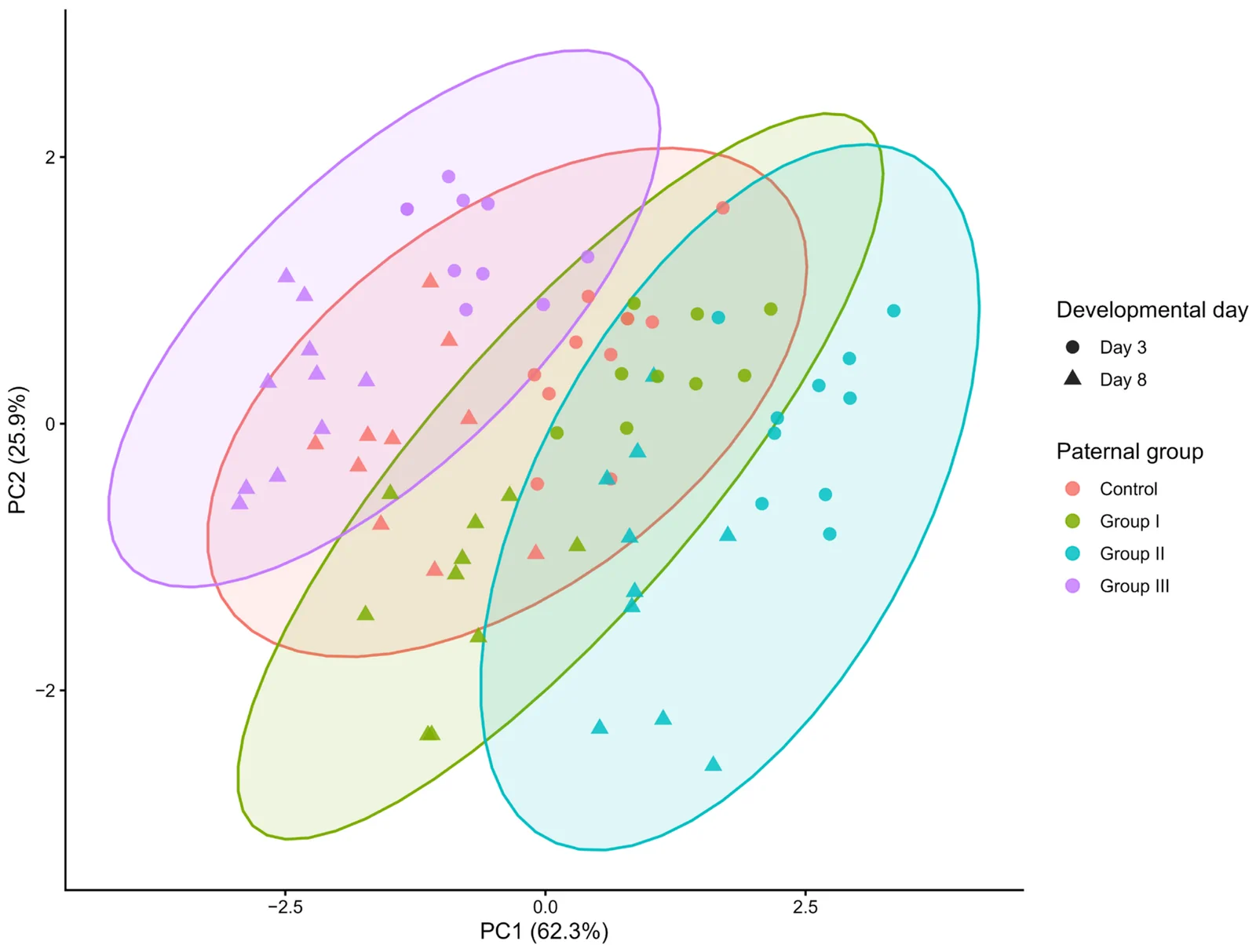
Principal component analysis of embryonic SIRT2, SIRT3, SIRT6, and SIRT7 expression profiles. Points represent individual experimental observations, colors indicate paternal reproductive groups, and point shapes indicate developmental day. Shaded areas represent 95% confidence ellipses calculated separately for each paternal group. PC1 and PC2 explained X% and Y% of the total variance, respectively.

Principal component analysis indicated differences in the distribution of embryo samples according to paternal reproductive group and oocyte source (Figure 4). Embryos derived from control bulls and Group III bulls occupied partly different regions of the PCA space, whereas embryos originating from Group I and Group II showed partial overlap with the remaining groups.

Embryos derived from heifer oocytes showed greater dispersion in the PCA space than embryos derived from cow oocytes. Because PCA is an exploratory method, the observed distribution should not be interpreted as a formal test of significant group separation.

### 2.5. Hierarchical Clustering of Embryonic Sirtuin Expression

**Figure 5:**
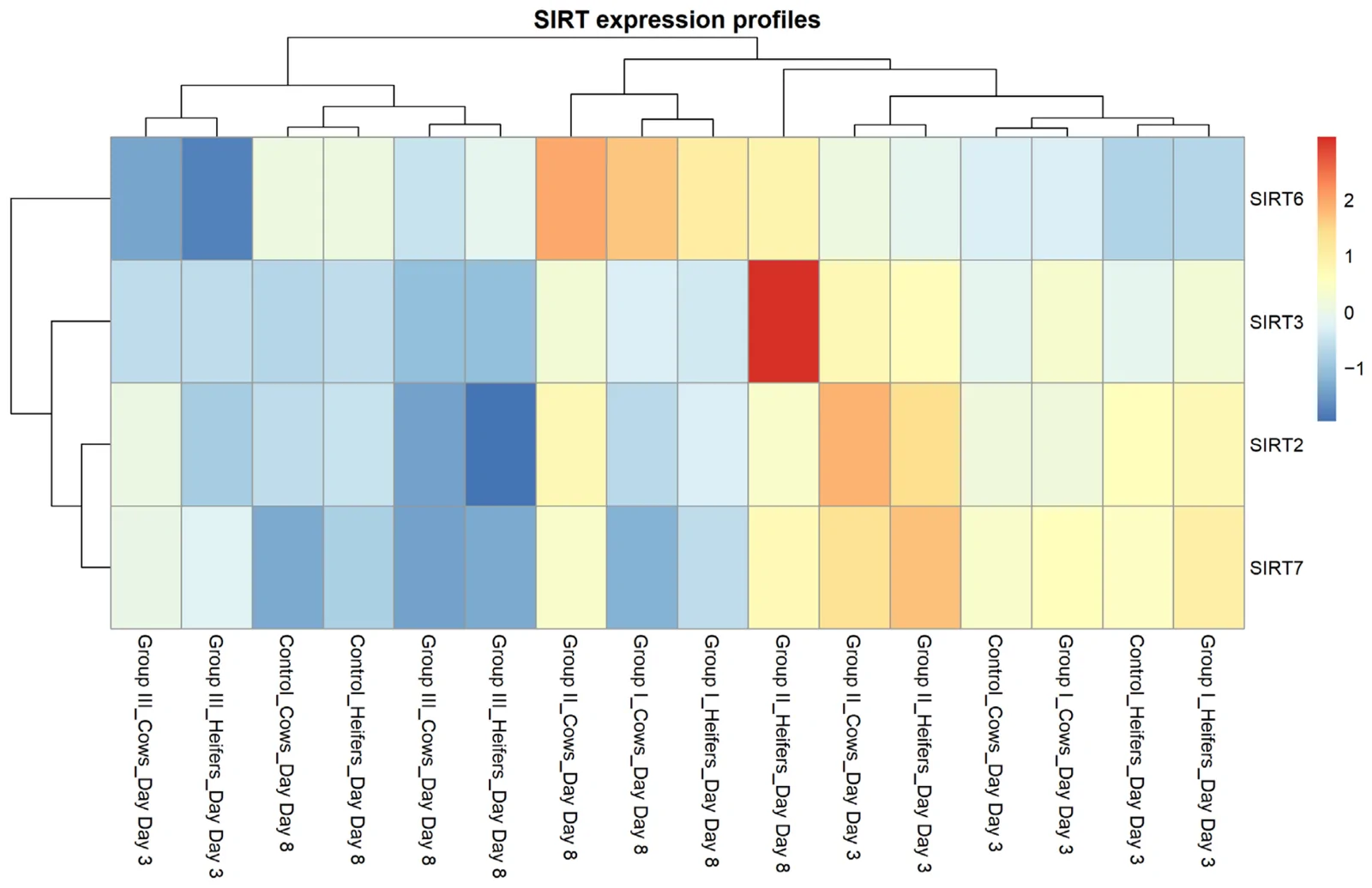
Hierarchical clustering heatmap of embryonic sirtuin expression profiles. Heatmap visualization was generated using standardized (Z-score) expression values of SIRT2, SIRT3, SIRT6, and SIRT7. Hierarchical clustering was performed using Euclidean distance and complete linkage. Rows represent individual sirtuins and columns represent experimental groups according to paternal reproductive status, oocyte source, and developmental stage.

Hierarchical clustering of standardized sirtuin expression values showed grouping patterns across paternal reproductive groups, oocyte sources, and developmental days (Figure 5). Observations from the Control and Group III groups tended to occupy different branches of the dendrogram, although overlap among experimental conditions remained. SIRT6 and SIRT7 displayed greater variation across grouped conditions than SIRT2 and SIRT3. Differences between Day 3 and Day 8 observations were also apparent. Because hierarchical clustering is exploratory, these patterns were not interpreted as formal evidence of group separation or coordinated molecular regulation.

### 2.6. Generalized Linear Mixed Model Analysis

Generalized linear mixed model analysis showed significant fixed-effect coefficients for paternal reproductive group and oocyte source (Table 1). Relative to the Control, Group III was associated with lower values of the modeled developmental outcome (Estimate = −1.148, *p* < 0.001), whereas Group I and Group II did not differ significantly from the Control (*p* > 0.05). Heifer-derived oocytes were also associated with lower values of the modeled developmental outcome than cow-derived oocytes (Estimate = −0.511, *p* < 0.001). No significant interactions involving Group I or Group II and oocyte source were detected (*p* > 0.05). A significant Group III × heifer-oocyte interaction was detected (Estimate = 0.544, *p* = 0.004).

## 3. Discussion

The present study identified associations between paternal reproductive phenotype, oocyte origin, preimplantation developmental outcomes, and embryonic sirtuin expression. Embryos generated using semen from Group III bulls showed lower developmental competence and lower SIRT6 and SIRT7 expression. These findings do not demonstrate a direct causal effect of libido disturbances or irregular semen collection. Rather, these characteristics should be regarded as phenotypic indicators of a broader reproductive condition that may coexist with endocrine, oxidative, metabolic, epigenetic, or sperm chromatin-related abnormalities.

Sperm DNA integrity, oxidative status, chromatin packaging, retained histones, DNA methylation, and sperm-borne small non-coding RNAs have previously been associated with fertilization, preimplantation development, and assisted-reproduction outcomes [9,10,11,12]. Experimental studies in cattle have further shown that sire identity and fertility status are associated with embryo developmental kinetics, embryonic stress responses, transcriptomic profiles, and molecular characteristics extending from sperm to the blastocyst stage [13,14,15]. The present results are compatible with the possibility that sperm-related molecular characteristics contributed to the observed embryo phenotypes. However, because these sperm variables were not included as covariates in the current statistical models, the present study cannot distinguish among the possible sperm-mediated mechanisms.

### 3.1. Functional Evidence for SIRT6 and SIRT7 in Embryonic Development

Published functional studies provide biological context for the associations observed in the present study. SIRT6 is a nuclear NAD^+^-dependent regulator associated with chromatin organization, DNA-damage responses, telomere stability, metabolic homeostasis, and adaptation to oxidative stress [16]. In preimplantation embryos cultured in vitro, reduced NAD^+^ availability was associated with impaired Sirt6-dependent telomere maintenance, mitochondrial reactive oxygen species accumulation, and epigenetic alterations. Supplementation with nicotinamide mononucleotide alleviated these abnormalities when functional Sirt6 was present, whereas this response was not maintained following conditional Sirt6 loss [17]. Consistent with the broader importance of NAD^+^ metabolism, experimental alterations of NAMPT activity have also been associated with changes in mitochondrial function and oxidative-stress status during early mouse embryo development [18]. The developmental relevance of telomere maintenance is additionally supported by evidence that blastocyst telomere length is associated with subsequent implantation success [19]. These findings provide functional evidence linking Sirt6 availability with telomere maintenance and cellular stress responses during preimplantation development.

Functional evidence concerning SIRT7 in preimplantation embryos is more limited. SIRT7 is a nuclear regulator associated with chromatin organization, genome maintenance, and cellular stress responses. Published experimental work examining the interaction between SIRT7 and *p53* during embryonic development places SIRT7 within developmental stress-response pathways [20]. However, these observations do not establish its specific function in bovine in vitro-produced embryos.

The present study differs fundamentally from these functional experiments because SIRT6 and SIRT7 protein abundance was quantified without genetic, pharmacological, or metabolic manipulation. Lower SIRT6 and SIRT7 expression in embryos generated using semen from Group III bulls, together with the observed correlations with developmental outcomes, are biologically consistent with the published functional evidence. Nevertheless, the current results do not demonstrate that either sirtuin mediates the association between paternal reproductive phenotype and embryo competence. SIRT6 and SIRT7 should therefore be interpreted as candidate molecular indicators associated with embryo developmental status rather than as confirmed causal regulators.

### 3.2. Oocyte Origin and Integrated Interpretation

Oocyte origin was primarily associated with the later developmental endpoint. Although fertilization, cleavage, and blastocyst formation did not differ significantly between cow- and heifer-derived oocytes, hatching rate was higher in embryos originating from cow oocytes. This pattern suggests that differences associated with oocyte origin may become more apparent during blastocyst expansion, trophectoderm function, and escape from the zona pellucida. However, the present study did not evaluate the cytoplasmic, mitochondrial, transcriptomic, or epigenetic characteristics of individual oocyte donors, and the mechanisms underlying this association remain unresolved.

The combined assessment of developmental outcomes, immunofluorescence-based sirtuin expression, correlation analysis, PCA, and hierarchical clustering provided complementary descriptions of the embryo phenotype. Across these analyses, SIRT6 and SIRT7 expression varied according to paternal reproductive group, oocyte origin, and developmental day. These observations support further evaluation of SIRT6 and SIRT7 as candidate molecular indicators of embryo competence but do not establish a regulatory or mediating role.

### 3.3. Limitations of the Study

Several limitations should be acknowledged. First, the present study was associative and did not include direct genetic, pharmacological, or metabolic manipulation of SIRT6 or SIRT7. Consequently, the current design cannot determine whether lower SIRT6 and SIRT7 expression precedes impaired embryo development, occurs as a consequence of developmental delay or cellular stress, or represents a compensatory response. The observed correlations should therefore not be interpreted as evidence that either sirtuin mediates or causes differences in embryo developmental competence. Follow-up studies should use controlled bovine in vitro embryo production models incorporating targeted loss- and gain-of-function approaches. These may include SIRT6 or SIRT7 inhibition and activation, gene silencing or overexpression, and supplementation with NAD^+^ precursors. Rescue experiments, in which an experimentally induced alteration is followed by restoration of sirtuin activity or NAD^+^ availability, would be particularly informative. Developmental endpoints should be evaluated together with mechanistic measures such as mitochondrial reactive oxygen species, DNA-damage markers, telomere length, apoptosis, total cell number, and inner-cell-mass and trophectoderm lineage markers.

Second, the semen samples originated from a previously characterized cohort of bulls, but semen quality, oxidative-stress indicators, DNA fragmentation, sperm chromatin status, and sperm sirtuin profiles were not included as covariates in the present models. The current analysis therefore cannot identify which specific sperm characteristic is associated with embryonic SIRT6 and SIRT7 expression or developmental outcomes. Future studies should integrate contemporaneously measured sperm functional and molecular indicators with embryo-level outcomes.

Third, oocytes were collected from slaughterhouse-derived ovaries. Although this approach is widely used in bovine in vitro embryo production studies, it does not allow complete control over donor physiological status, follicular dynamics, metabolic condition, or ovarian reserve. These uncontrolled maternal characteristics may have contributed to the observed variability, particularly at later stages of preimplantation development.

## 4. Materials and Methods

The experiment was carried out as part of routine activities during the current semen production at the reproductive station and did not require approval from the ethics committee.

### 4.1. Animals and Experimental Group

The study was carried out using semen samples obtained from Holstein–Friesian bulls maintained under uniform environmental and nutritional conditions at an artificial insemination station. Bulls were assigned to four experimental groups according to age, reproductive performance, and libido status: control bulls aged approximately 24 months characterized by regular semen collection and normal libido (control), sexually maturing bulls aged approximately 14 months (Group I), mature breeding bulls older than 3 years with regular semen collection (Group II), and bulls older than 2 years presenting libido disorders and irregular semen collection (Group III). Each experimental group consisted of eight animals.

Bovine ovaries used for oocyte collection were obtained from Holstein–Friesian females at a local slaughterhouse. Donor females were classified into two groups according to reproductive status and age: mature cows and sexually mature heifers before the first calving. Cumulus–oocyte complexes derived from both donor groups were used for subsequent in vitro embryo production procedures.

### 4.2. Semen Collection and Evaluation

The ejaculates used in the present study originated from the same bull cohort that had previously undergone comprehensive semen characterization, including computer-assisted sperm analysis (CASA), flow cytometric assessment of sperm quality, oxidative stress assessment, and sperm sirtuin profiling (Supplementary Material S1). These analyses were performed as part of an independent experiment designed to investigate molecular determinants of semen quality and are therefore not reported here.

For the present study, aliquots of the previously characterized ejaculates were used exclusively for in vitro fertilization procedures and subsequent embryo culture. The current experiment was designed independently and focused on embryo development competence and embryonic sirtuin expression. No semen quality, oxidative stress, or sperm sirtuin data generated in the previous experiment were re-analyzed or reproduced in the present manuscript, except for descriptive information necessary to characterize the biological material.

The present study did not include any evaluation of semen quality parameters, oxidative status, or sperm sirtuin expression. These variables were previously evaluated in an independent experiment and were not incorporated into the statistical analyses reported herein. Consequently, these variables were not treated as covariates or candidate mediators in the present models. The current study was designed to compare embryo developmental and molecular outcomes according to predefined paternal reproductive groups rather than to perform a post hoc mediation analysis of sperm characteristics.

### 4.3. Oocyte Collection and In Vitro Maturation

The bovine ovaries were obtained from a local slaughterhouse and transported to the laboratory in 2 h in sterile physiological saline supplemented with antibiotics at approximately 37 °C. Cumulus–oocyte complexes (COCs) were aspirated from ovarian follicles and morphologically selected under a stereomicroscope. Only oocytes surrounded by compact multilayered cumulus cells and homogeneous cytoplasm were included in the study. Oocytes were divided according to donor origin into two experimental groups: cows and heifers. The selected COCs were matured in vitro in maturation medium under humidified atmospheric conditions containing 5% CO_2_ at 38.5 °C. In vitro maturation procedures were performed according to protocols previously described in our previous study [21], with minor modifications.

### 4.4. In Vitro Fertilization and Embryo Culture

After in vitro maturation, oocytes were subjected to in vitro fertilization using frozen-thawed semen originating from bulls assigned to the respective experimental groups. Sperm preparation and IVF procedures were performed according to standard bovine embryo production protocols. Gametes were co-incubated under controlled culture conditions at 38.5 °C in a humidified atmosphere containing 5% CO_2_. After fertilization, the presumptive zygotes were denuded of the cumulus cells and cultured in embryo culture medium under standard incubation conditions until day 8 of development. Embryo culture was carried out under mineral oil in a humidified atmosphere containing 5% CO_2_, 5% O_2_, and 90% N_2_, as we described earlier [21].

### 4.5. Embryonic Developmental Assessment

Embryo developmental competence was evaluated during selected stages of preimplantation development according to standard bovine embryo assessment criteria [22,23]. The fertilization rate was evaluated on day 1 following IVF based on the presence of presumptive zygotes; the cleavage rate was evaluated on day 3, whereas blastocyst formation and hatching rates were assessed on Day 8 of embryo culture. Developmental outcomes were expressed as percentages relative to the number of inseminated oocytes. Embryos were morphologically evaluated under an inverted fluorescence microscope (MAGUS V700, Levenhuk, Tampa, FL, USA) and developmental endpoints were compared among experimental groups that differed in paternal reproductive status and oocyte origin [22,23].

### 4.6. Immunofluorescence Analysis of Embryonic Sirtuins

The embryonic expression of SIRT2, SIRT3, SIRT6, and SIRT7 was evaluated by immunofluorescence analysis. Embryos were fixed in 4% paraformaldehyde at room temperature, washed in phosphate-buffered saline, permeabilized with Triton X-100, and blocked in bovine serum albumin solution to reduce nonspecific antibody binding.

Embryos were subsequently incubated overnight, 12 h at 4 °C, with the following primary antibodies: mouse monoclonal anti-SIRT2 antibody (Proteintech Group, Rosemont, IL, USA; catalog no. 66410-1-Ig; RRID: AB_2881782; dilution 1:150; lot no. 10019977), rabbit polyclonal anti-SIRT3 antibody (Thermo Fisher Scientific, Waltham, MA, USA; catalog no. PA5-117095; RRID: AB_2901725; dilution 1:150; lot no. 79763522), rabbit polyclonal anti-SIRT6 antibody (Thermo Fisher Scientific; Waltham, MA, USA; catalog no. PA5-17215; RRID: AB_10977684; dilution 1:100; lot no. 79763910), or FITC-conjugated rabbit polyclonal anti-SIRT7 antibody (FabGennix, Frisco, TX, USA; catalog no. SIRT7-FITC; RRID not available; dilution 1:150; lot no. 79767531). Following primary antibody incubation, embryos were washed thoroughly in phosphate-buffered saline. Embryos labeled with the unconjugated SIRT2, SIRT3, or SIRT6 primary antibodies were incubated with the appropriate fluorophore-conjugated secondary antibodies for 1 h at room temperature in the dark. An anti-mouse IgG secondary antibody was used for SIRT2 detection, whereas an anti-rabbit IgG secondary antibody was used for SIRT3 and SIRT6 detection. Because the anti-SIRT7 primary antibody was directly conjugated to FITC, no secondary antibody was used for SIRT7 detection. Nuclear counterstaining was performed using DAPI for 10 min at room temperature in the dark.

Negative controls were processed under the same experimental conditions, except that the primary antibody was omitted. Fluorescence images were acquired using an inverted fluorescence microscope under identical exposure, magnification, illumination, and image-acquisition settings for all experimental groups. Mean fluorescence intensity was quantified using ImageJ software v. 1.52s (National Institutes of Health, Bethesda, MD, USA). For each embryo, the region of interest was delineated manually, and the mean fluorescence intensity was measured after subtraction of the corresponding background fluorescence. Background-corrected fluorescence values were further normalized against the fluorescence recorded in the respective negative controls.

### 4.7. Statistical Analysis

All statistical analyses were performed using R software v. 4.5.2 (R Foundation for Statistical Computing, Vienna, Austria). Data distributions were initially assessed using the Shapiro–Wilk test, and descriptive statistics were expressed as mean ± standard deviation (SD).

Embryo developmental outcomes, including fertilization, cleavage, blastulation, and hatching rates, were analyzed using generalized linear mixed-effects models with a binomial distribution and logit link function implemented in the lme4 package. Paternal reproductive group (Control, Group I, Group II, and Group III), oocyte source (cows versus heifers), and their interaction were included as fixed effects, whereas experimental replicate was included as a random effect. Pairwise post hoc comparisons were performed using estimated marginal means with Tukey adjustment implemented in the emmeans package.

Expression levels of SIRT2, SIRT3, SIRT6, and SIRT7 were analyzed using linear mixed-effects models. Paternal reproductive group, oocyte source, developmental day (Day 3 versus Day 8), and all two-way and three-way interactions were included as fixed effects, whereas experimental replicate was included as a random intercept. Models were fitted using restricted maximum likelihood estimation. The statistical significance of fixed effects was assessed using Type III analysis-of-variance tables with Satterthwaite approximation of denominator degrees of freedom, implemented in the lmerTest package. Sum-to-zero contrasts were used for categorical predictors. Significant effects were followed by comparisons of estimated marginal means with Tukey adjustment using the emmeans package.

Associations between embryo developmental outcomes and embryonic sirtuin expression were evaluated using two-sided Spearman’s rank correlation coefficients. All prespecified pairwise associations between the measured developmental outcomes and SIRT2, SIRT3, SIRT6, and SIRT7 expression were calculated and displayed. No minimum absolute correlation coefficient, significance-based screening threshold, or other data-driven criterion was applied to select correlations for presentation; therefore, no correlations were excluded from the correlation matrix based on their magnitude or *p* value. The correlation analysis was treated as exploratory.

Principal component analysis was performed using the prcomp function and standardized expression values of SIRT2, SIRT3, SIRT6, and SIRT7. Before PCA and hierarchical clustering, each sirtuin variable was standardized according to:

$$Z_{ij}=\frac{x_{ij}-{\bar{x}}_{j}}{s_{j}}$$

where x_ij_ represents the expression value for observation i and sirtuin j, x_j_ represents the arithmetic mean expression value for sirtuin j across all included observations, and s_j_ represents the corresponding sample standard deviation. Consequently, each standardized variable had a mean of zero and a standard deviation of one. The same standardized dataset was used for PCA and hierarchical clustering. Ninety-five percent confidence ellipses were calculated separately for each paternal reproductive group and superimposed on the PCA score plot to visualize group boundaries and overlap.

Hierarchical clustering and heatmap visualization were performed using Euclidean distance and complete-linkage clustering implemented in the pheatmap package. Model diagnostics for generalized linear mixed-effects models were assessed using simulation-based residual diagnostics implemented in the DHARMa package, including tests of residual distribution and dispersion. Data visualization was performed using the ggplot2 package (version 4.0.2). Statistical significance was defined as *p* < 0.05.

#### Relationship with Previous Work

The semen samples used in this study originated from a biological collection that had been characterized previously within a separate experimental framework (Supplementary Material S1). The previous experiment addressed the functional and molecular characteristics of sperm, whereas the present study investigated post-fertilization developmental outcomes and embryonic sirtuin expression. Although the biological material was shared between the experiments, their hypotheses, endpoints, datasets, and statistical analyses were distinct. The previous sperm measurements were not predefined as mediators or covariates in the present study and therefore were not incorporated post hoc into the current models.

## 5. Conclusions

The present study found that paternal reproductive phenotype and oocyte origin were associated with bovine preimplantation developmental outcomes and embryonic sirtuin-expression profiles. Embryos generated using semen from Group III bulls showed lower developmental competence and lower SIRT6 and SIRT7 expression, whereas cow-derived oocytes were associated with higher hatching rates than heifer-derived oocytes. Among the analyzed sirtuins, SIRT6 showed the strongest positive correlation with hatching. These findings identify embryonic SIRT6 and SIRT7 as candidate molecular indicators associated with embryo competence in bovine in vitro embryo production systems. Because the study did not include functional manipulation of these proteins, their causal, secondary, or compensatory roles remain unresolved. Controlled intervention studies are required to determine the biological significance and direction of these associations.

## Figures and Tables

**Table 1:** Fixed-effect estimates from the generalized linear mixed model (GLMM) evaluating the effects of paternal reproductive status, oocyte source, and their interaction on embryo developmental competence.

Effect	Term	Estimate	SE	*z* Value	*p* Value
Fixed	Intercept	0.878	0.105	8.350	<0.001
Fixed	Male Group I	0.107	0.152	0.706	0.480
Fixed	Male Group II	0.068	0.146	0.462	0.644
Fixed	Male Group III	−1.148	0.144	−7.957	<0.001
Fixed	Oocyte source: Heifers	−0.511	0.137	−3.740	<0.001
Fixed	Group I × Heifers	−0.142	0.195	−0.731	0.465
Fixed	Group II × Heifers	0.038	0.192	0.198	0.843
Fixed	Group III × Heifers	0.544	0.189	2.878	0.004

## Data Availability

The data presented in this study have been deposited in the Research Data Repository of the Wrocław University of Environmental and Life Sciences and are publicly available at: [https://doi.org/10.57755/7an6-cc98; https://doi.org/10.57755/t18n-5278].

## Supplementary Materials

The following supporting information can be downloaded at: https://www.mdpi.com/article/10.3390/ijms27167115/s1.
