# Supplementary material for: Altered Embryonic SIRT6 and SIRT7 Expression Is Associated with Impaired Developmental Competence and Oocyte-Dependent Embryo Quality in Embryos Derived from Subfertile Males
Source: Int J Mol Sci. 2026 Aug 8;27(16):7115. doi: 10.3390/ijms27167115 (PMC13512963; doi:10.3390/ijms27167115)
Supplement: Supplementary file 1 [file ijms-27-07115-s001.zip › ijms-4452117-supplementary.pdf]

Supplementary Material S1. Previous characterization of semen samples used for IVF

The ejaculates used for in vitro fertilization originated from a cohort of bulls previously subjected to semen quality characterization in an independent experiment. The characterization included assessment of sperm motility, viability, DNA integrity, oxidative status, and sperm sirtuin expression.

These measurements were performed solely to characterize the biological material and are not analysed or reported in the present manuscript. Detailed experimental procedures and results are described elsewhere (Kowalczyk et al., manuscript under review).
